# Engineering of Synthetic Microbial Consortia for Sustainable Management of Wastewater and Polyethylene Terephthalate: A Comprehensive Review

**DOI:** 10.3390/ijms262311623

**Published:** 2025-11-30

**Authors:** Yiqun Zhou, Muhammad Zeeshan Ul Haq

**Affiliations:** 1 Faculty of Science, The University of Hong Kong, Hong Kong SAR 999077, China; zhouyiqun2025@163.com; 2 Spice and Beverage Research Institute, Chinese Academy of Tropical Agriculture Science, Wanning 571533, China; 3 School of Breeding and Multiplication (Sanya Institute of Breeding and Multiplication), School of Tropical Agriculture and Forestry, Hainan University, Sanya 572025, China

**Keywords:** synthetic microbial consortia, wastewater treatment, polyethylene terephthalate (PET), CRISPR-Cas9, machine learning, quorum sensing, cross-feeding, enzyme engineering

## Abstract

Plastic pollution and wastewater have become the leading environmental concerns due to their harmful effects on human health and pose a severe threat to the biosphere. Polyethylene terephthalate (PET) is one of the most widely used plastics worldwide, but it is resistant to natural degradation. Additionally, the complex pollutants in wastewater demand advanced remediation strategies. Although physicochemical methods are commonly used for PET degradation and wastewater treatment, bioremediation with microorganisms offers a greener and more eco-friendly alternative. This review focuses on the molecular mechanisms and engineering of synthetic microbial consortia (SMC) for the bioremediation of wastewater and PET plastics. It examines the rational design of SMCs, utilizing both bottom-up and top-down methods, and emphasizes the importance of quorum sensing and metabolite cross-feeding in maintaining the stability and functionality of the consortium. Furthermore, the review critically assesses how CRISPR-Cas9 enables precise genome editing for robust pathway engineering and stress resilience, while Machine Learning provides predictive models to optimize consortium composition and function, thereby advancing SMC capabilities for both applications. These developments highlight SMC as a promising, eco-friendly, and efficient biological platform to tackle wastewater challenges and plastic pollution simultaneously.

## 1. Introduction

In recent years, water pollution has become a significant threat to public health and a global challenge for environmental restoration. Industrial and domestic untreated effluents are one of the key aspects of contaminating water bodies [1], affecting human health and the ecosystem. It is estimated by the UN World Water Development Report of 2024 that 80% of the water is released into the environment without adequate treatment, especially in developing countries [2]. The UNEP Global Environment Outlook (2024) reported that pharmaceuticals, microplastics, and personal care products pollute more than 60% of freshwater bodies worldwide [3]. In the modern era, the world is going through the Plastic Age, and plastic pollution has emerged as one of the most significant environmental issues because of its extensive use in daily life. Despite reducing its impact through recycling and waste management, the low biodegradability of plastics has led to a growing concern caused by their detrimental effects on the natural ecosystem. Although annual single-use plastic generation is still growing exponentially, it was estimated that only 18% of the plastic waste has been recycled worldwide. The world’s plastic production is expected to reach 34 billion tons by 2050 [4], resulting in 12,000 million tons of plastic contaminating the environment [5]. Additionally, nearly 6.4 million tons of waste enter the ocean every year.

One of the most used plastics in the world is polyethylene terephthalate (PET). This synthetic polymer exhibits excellent durability and low biodegradability, which can harm human health and the aquatic ecosystem [6]. The inherent resistance of PET is a direct consequence of its molecular structure, characterized by aromatic rings and strong ester bonds that form a highly crystalline and hydrophobic material. Consequently, waste management has become a critical global issue, necessitating the development of sustainable and innovative approaches to mitigate plastic in the wastewater. Traditional methods, including physical cycling, thermal treatment, chemical recycling, biological depolymerization, and upcycling, have already been proposed and investigated for efficient PET-processing [7]. Compared with traditional physical and chemical treatments, bioremediation offers cleaner and more economical advantages [8,9]. Instead, microbial consortia can degrade various pollutants into non-toxic or less-toxic compounds [10]. Research on bioremediation by SMC is practical and has been applied in some cases, as they can degrade PET [11,12] and sewage pollutants [13].

Microbial consortia exhibit exceptional abilities to degrade complex compounds, such as starch and cellulose, in sewage, which single bacterial systems struggle to break down. Some compounds are difficult to decompose due to their complex structures; however, certain strains can break these down into small-molecule sugars that other strains can then utilize as carbon sources [14]. In addition to natural microbial consortia, synthetic microbial consortia (SMC) are designed to distribute the desired multiple catalytic enzyme expression pathways more efficiently among different strains. The composition of SMC is simple, the division of labor is clear [15], and it can be further modified for different target products. SMC not only carry out the desired functions but also sustains cell growth in a stable and robust way. The degradation efficiency of complex compounds can be improved by cross-feeding between bacteria to eliminate feedback inhibition and remove products or by-products. The adaptability and stability of synthetic microbial consortia systems (SMCs) [16] are enhanced when microbes with different functions are constructed, and their interactions help maintain a dynamic balance in varying environmental conditions.

Modern genome editing (CRISPR-Cas9) is an effective tool to engineer the microbes in SMCs to degrade plastic more effectively and selectively [17]. The division of metabolic pathways in the SMCs eliminates cross-reactions and lowers the metabolic load on individual cells. CRISPR-Cas9 makes it possible to precisely edit genes that regulate metabolic processes, stress tolerance, and pollutant breakdown. Subsequently, the functional roles and collaborative dynamics between single strains are increased [18]. Research has highlighted that CRISPR has also been utilized to engineer strains by creating specific consortia that can effectively decompose various hydrocarbons or eliminate heavy metals in wastewater [19].

**Figure 1:**
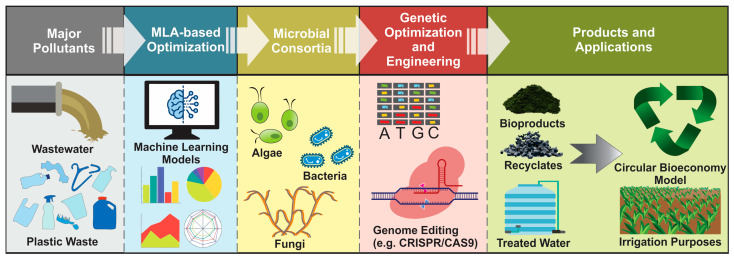
Schematic illustration of bioremediation of wastewater and plastic using microbial consortia, highlighting the integration of molecular tools.

Moreover, machine learning (ML) techniques are becoming increasingly common for predicting microbial interactions, metabolic outputs, and optimal strain combinations using extensive biological datasets [20]. ML helps make communities more stable, productive, and resilient by stimulating community functioning and guiding experimental work. ML also facilitates the CRISPR-guided RNA optimization, which significantly enhances the accuracy of engineering through improvement of the precision of editing and minimizing off-target effects [21]. Using CRISPR-Cas9 and ML, it is possible to make versatile SMCs [22] that can be utilized to treat wastewater and plastic degradation. The schematic illustration of the overall theme of this review is shown in Figure 1.

This review provides an in-depth examination of the molecular mechanisms underpinning SMCs’ function, from enzymatic degradation pathways to engineered microbial interactions. We detail the construction of SMCs through top-down and bottom-up approaches and their potential in wastewater treatment and the degradation of PET plastic. It also emphasizes the importance of metabolic division of labor, cross-feeding, and quorum sensing in SMCs to enhance pollutant breakdown and stability in various scenarios. Furthermore, CRISPR/Cas9 and advanced machine learning techniques are also thoroughly reviewed for their innovative methods to precisely engineer strains and optimize consortia at the molecular level. Lastly, recent developments in environmental adaptability, strain engineering, and microbial interactions, as well as future directions for enhancing SMCs design and deployment for practical and sustainable bioremediation, are also discussed.

While previous reviews have comprehensively covered the genetic engineering of microalgae for specific wastewater streams [23], this review provides a distinct and integrated perspective on the engineering of multi-kingdom synthetic consortia for the simultaneous bioremediation of both wastewater and PET, highlighting the convergence of advanced molecular tools and computational design.

## 2. Role of Molecular Mechanisms of Specific Microbes in Bioremediation

Specific microorganisms play vital roles in bioremediation due to their diverse metabolic capabilities, which enable them to mitigate environmental pollutants. This section reviews the key enzymes, biochemical pathways, and molecular mechanisms that enable bacteria, fungi, and microalgae to degrade PET and perform wastewater remediation. It provides the foundational knowledge for constructing effective SMCs. Various microorganisms (microalgae, bacteria, and fungi) are capable of plastic degradation, wastewater treatment, and absorption of heavy metals. Typically, these microorganisms can adhere to the PET surface and utilize it as a primary source of carbon and energy for their growth. Beyond PET degradation, many organisms contribute significantly to wastewater treatment by breaking down organic pollutants, removing nutrients, and decomposing emerging contaminants through diverse enzymatic reactions [24]. They also detoxify or immobilize heavy metals, such as cadmium, lead, and mercury, through biosorption, bioaccumulation, and enzymatic transformation, thereby reducing their bioavailability and toxicity.

### 2.1. Bacteria in Bioremediation

Bacteria have great potential to break down organic pollutants in wastewater and degrade synthetic plastics. The initiation of biodegradation involves the breakdown of high-molecular-weight long-chain polymers. Certain bacteria synthesize PETases, cutinases, and laccases to target and degrade synthetic polymers. For example, *Ideonella sakaiensis* produces PETase enzymes that break down PET into mono(2-hydroxyethyl) terephthalic acid [25]. These monomers are further processed by MHETase enzymes into smaller compounds. Cutinases are a unique group of esterases that have great potential to hydrolyze PET. *T. cellulosilytica*, *T. fusca*, and *T. alba* [26] are well-studied strains that produce cutinases. Beyond cutinases, pollutants are also reported to be degraded by oxygenases, hydrolases, and depolymerizing enzymes. These enzymes are primarily produced by *Bacillus*, *Rhodococcus*, and *Escherichia* [27]. Other studies [28] reported that *Pseudomonas aeruginosa* and *Bacillus megaterium* also produce depolymerase enzymes, which degrade PET polymer chains into less toxic compounds. Moreover, Streptomyces scabies is an emerging candidate for hydrolyzing a range of natural and synthetic polymers. It produces the Sub1 enzyme that is effective in breaking down p-nitrophenyl esters. Sub1 also releases the terephthalic acid (TPA) [29], which exhibits a promising degradation ability on the synthetic polymer PET. Bacteria are also known to be fundamental to wastewater treatment due to their ability to degrade a wide range of organic pollutants through various metabolic pathways. Bacteria metabolize complex organic matter into simpler compounds, producing biomass and regenerating oxygen in the process [30]. They also play a significant role in heavy metal bioremediation through mechanisms such as biosorption, bioaccumulation, enzymatic transformation, and bioprecipitation. For example, *Bacillus cereus* has shown removal efficiencies of up to 81% [31] for cadmium and 40% for lead at specific concentrations.

### 2.2. Fungi in Bioremediation

Fungi can break down PET into low molecular weight oligomers or monomers [32] such as bis(2-hydroxyethyl)terephthalate (BHET) and mono(2-hydroxyethyl) terephthalate (MHET). The monomeric structural units of PET are linked by ester bonds, which are easily hydrolyzed by fungal enzymes. The main classes of enzymes involved in plastic biodegradation are hydrolases and oxidoreductases. The main hydrolytic enzymes involved in PET degradation are cutinases, lipases, and carboxylesterases. Esterases cleave the ester bonds and assist the surface modification of the target PET [33]. Lipases are widely known for their catalytic hydrolysis of PET fabrics to some extent, which enhances their wettability, and the interfacial activation phenomenon characterizes them. As discussed earlier in the previous Section 2.1, cutinases are lipolytic esterolytic enzymes that cleave PET through the hydrolysis of ester bonds and release intermediate products that can subsequently be metabolized to degrade the pollutants. The intermediate products include bis(2-hydroxyethyl) terephthalate (BHET), mono(2-hydroxyethyl) terephthalate (MHET), terephthalic acid (TPA), and ethylene glycol (EG). They exhibit the most efficient depolymerization capabilities of PET among other enzymes. Other strains, such as *Aspergillus*, *Ganoderma*, and *Phanerochaete*, also produce oxidative enzymes to degrade PET by secreting extracellular laccases and peroxidases [34]. Apart from PET degradation, fungi have diverse enzymatic capabilities and unique physiological features that make them key players in wastewater treatment and heavy metal bioremediation. Other filamentous fungi secrete a wide variety of extracellular enzymes (lignin peroxidase, manganese peroxidase, laccase, and cellulases) that catalyze the degradation of complex organic pollutants in wastewater [35]. *Phanerochaete chrysosporium* and *Trametes versicolor* are particularly effective in degrading micropollutants by oxidizing their complex molecular structures through oxidative enzymatic reactions. Fungi can also be applied as whole-cell cultures or as enzyme extracts in both laboratory and pilot-scale bioreactors [36].

### 2.3. Microalgae in Bioremediation

Microalgae have potential in bioremediation through biosorption, bioaccumulation, and biodegradation. Biosorption involves the binding of heavy metals, organic compounds, and dyes to the surface of microalgae. Functional groups on the cell wall of microalgae and extracellular polymeric substances (EPS) interact with pollutants and facilitate biosorption [37]. Microalgal cells can also uptake contaminants into the cytoplasm through facilitated transmembrane diffusion and energy-dependent active uptake [38] to remove them from the environment. This uptake induces stress in microalgae and activates the antioxidative defenses. For example, *Dunaliella salina* upregulates arsenic transporters when exposed to arsenic and performs detoxification by converting it into less harmful organic arsenicals [39]. Certain microalgae species exhibit selective biodegradation capabilities by targeting specific contaminants while leaving beneficial components in the water unharmed. Researchers evaluated that *Chlorella vulgaris* and *Phaeodactylum tricornutum* can degrade iohexol and sulfadimethoxine, respectively, achieving final removal rates of 40–59% [40]. Other researchers also reported the potential of *Chlorella vulgaris* and *Scenedesmus* in reducing the nitrogen and phosphorus in wastewater [41]. Although microalgae cannot directly degrade plastic, recent advances in genome editing have made it possible to genetically engineer microalgal species to produce enzymes that can break down plastic. Recent studies [42] have investigated the ability of genetically modified microalgae (*Chlamydomonas reinhardtii*) to express PETase enzymes. The PETase from *Ideonella sakaiensis* was constitutively expressed in the chloroplast of *C. reinhardtii*, which can actively depolymerize PET and post-consumer plastics. Another study [43] highlighted that microalgae oxidize low-molecular-weight molecules, followed by the degradation of larger molecules for plastic bioremediation and mitigating heavy metals [44]. Algae can bind heavy metals through cell binding and intracellular processes involving metallothioneins and phytochelatins [45].

While Section 2.1, Section 2.2 and Section 2.3 have outlined the roles of individual microbial groups in bioremediation, natural microbial consortia leverage synergistic interactions among bacteria, fungi, and microalgae to degrade complex pollutants more effectively than single strains. For instance, bacteria provide rapid enzymatic degradation, fungi offer robustness against recalcitrant compounds, and microalgae enhance sustainability through photosynthesis and biosorption, with consortia, such as microalgae-bacteria systems, achieving more than 90% chemical oxygen demand (COD) and nutrient removal [46]. These comparative advantages are summarized in Table 1, which illustrates their relevance in complementary mechanisms for wastewater and PET remediation. However, natural consortia often suffer from limitations, such as unpredictable interactions, competitive dominance by species, and reduced efficiency under varying conditions. These limitations can lead to inconsistent outcomes [46,47]. These challenges highlight the need for engineered synthetic microbial consortia (SMCs) to achieve greater stability and targeted functionality.

## 3. Synthetic Microbial Consortia Construction

As discussed in Section 2, Bioremediation relies on the metabolic capabilities of microorganisms to degrade pollutants, but natural systems often lack the precision and efficiency needed for complex contaminants like those in wastewater and PET plastics. SMCs bridge this gap by engineering defined microbial communities that enhance bioremediation through optimized interactions, such as quorum sensing and cross-feeding, division of labor, and metabolic pathways [49]. SMCs are particularly relevant for plastic and wastewater bioremediation, as they can be tailored to express specific enzymes (e.g., PETase and MHETase) and achieve higher degradation rates, stability, and adaptability compared to single strains or natural consortia, paving the way for scalable, eco-friendly solutions. This section explores the construction of SMCs, focusing on top-down and bottom-up approaches, quorum sensing, and cross-feeding to enable their effective design and implementation in these applications.

### 3.1. Top-Down and Bottom-Up Approaches

The top-down approach is a classical method that utilizes a natural microbial consortium. This approach involves the manipulation of environmental variables such as pH, temperature, light, and oxygen levels to design the function of SMCs and enable them to perform the desired biological process. To optimize the construction of SMCs, it is essential to understand the system’s inputs and outputs, as well as the biotic and abiotic processes involved. Microbial consortia can be sourced from different natural and engineered microbial ecosystems such as soil, wetlands, compost, activated sludge, and anaerobic digesters [47]. The most used method in top-down approaches is artificial enrichment [46]. Artificial enrichment promotes the growth of the microbial population with the desired features over other populations through repeated dilutions under controlled conditions [50]. The majority of the microbial consortia that degrade complex compounds are constructed with the top-down approach. Although the conventional top-down approach is widely successful [51], limited knowledge of microbial interactions and cellular processes can potentially limit the microbial populations with desired features. This leads to a lower product yield. Therefore, the top-down approach is limited due to unpredictable outcomes, undesirable competitive reactions, and challenges in linking the taxonomic identity of microbes with function [52]. To overcome these limitations of the top-down approach, bottom-up strategies have been developed that allow for more precise control over consortium composition and function.

Recent developments in synthetic biology have allowed researchers to create bottom-up approaches by engineering the metabolic network and microbial interactions within SMCs to achieve specific cellular characteristics [53]. This involves obtaining the genomes of individual members of the SMCs and then reconstructing the metabolic networks [54]. The metabolic reactions of individual populations can be separated into compartments [55] to model the metabolic fluxes using principles of optimality. These bottom-up tools are being used to design microbes with targeted characteristics in a systematic manner [56]. To enhance ecosystem function and stability, distributed pathways, modular species interactions, and spatiotemporal organization can be designed using these approaches [57]. Consequently, the implementation of such designs into systems containing non-model organisms would require more understanding of their metabolism, behaviors, and principles of their interactions [58]. The main problems that researchers encounter while constructing SMCs include selecting a suitable chassis strain and dividing degradation pathways within the consortia [59]. These problems must be addressed to choose the strain that exhibits suitable performance within consortia and high mass transfer efficiency.

### 3.2. Quorum Sensing

The most widely used systems to synthetically design and sustain SMCs are quorum sensing (QS) and cross-feeding. QS is a widely used mechanism for inter-species and intra-species communication to regulate collective behaviors and coordinate complex behavior in SMCs [60]. Several QS systems have been identified in environmental microorganisms, such as lux, esa, las, tra, rpa, rhl, and cin [61]. These are critical in regulating the process of intercellular communication and coordination. Such systems regulate a large variety of bacterial characteristics such as genetic phenotypes, biofilm formation, and the promotion or inhibition of specific functions. QS systems also regulate symbiosis, mutualism, and virulence within SMCs [62]. For example, Researchers have engineered the esaR promoters with an additional EasR binding site to adjust QS-dependent gene expression [63]. With this development, a single QS signal can be used to regulate multiple genes, offering new ways to control microbial behaviors in SMCs. Nevertheless, one challenge in microbial consortia is that many QS systems are not entirely orthogonal. But recently, two engineered QS systems (rpa and tra) were studied to be completely orthogonal [64]. Additionally, a sophisticated QS circuit was designed and used to autonomously and temporarily control three metabolic fluxes involved in a pathway [65].

### 3.3. Cross Feeding

While quorum sensing provides the communication network for coordinating population-level behavior, cross-feeding represents the fundamental metabolic exchange that sustains the consortium. These two mechanisms are deeply interdependent; QS can regulate the production of cross-fed metabolites, and the metabolic state of cells can influence QS signal production, creating integrated feedback loops that maintain community stability and function. Cross-feeding is a key microbial interaction in nature that can enhance the growth of both partners, leading to more sustainable and faster growth compared to when they grow alone. This interaction is typically considered a mutualistic interaction of the microbes in SMCs [66]. For example, syntrophy is an environmental consortium based on mutualism, where one organism continuously utilizes a compound produced by another organism [67]. Cross-feeding typically involves either single-metabolite or multiple-metabolite cross-feeding [68]. Cross-feeding based on a single metabolite is mostly common in small consortia. In single metabolite cross-feeding, auxotrophs form complex interactions between microbes, such as amino acids [69]. Such integrations support stability and resilience because they make community members dependent metabolically. But in large microbial consortia, it is insufficient to support the normal growth of all strains, which restricts their strength and stability. Therefore, cross-feeding based on multiple metabolites is becoming more feasible for degrading complex compounds. It is used to enhance the interaction among microbial entities involving many strains within a microbial consortium. Under this approach, the selection of the right metabolic pathways is important, which involve multiple metabolites essential for cell growth to move across cell membranes. For instance, in a PET-degrading consortium, one strain might be engineered to consume TPA. In contrast, another consumes EG, with both strains cross-feeding essential nutrients or cofactors to each other.

## 4. Synthetic Microbial Consortia in Wastewater Treatment

The large amount of effluent discharged by industries, residences, and public facilities is becoming a serious global issue with modern urbanization. This type of sewage is primarily biodegradable and can be recycled into a circular bioeconomy to produce valuable bioproducts. Traditional treatment systems are typically expensive and require substantial amounts of energy. But they often fail to address all the challenges associated with sewage [70]. The complexity of wastewater, which contains diverse organic pollutants, nutrients, heavy metals, and emerging contaminants, presents a challenge that often exceeds the metabolic capabilities of any single microbial strain.

SMCs are the most efficient and relatively clean method for treating sewage and producing bioproducts over monoculture approaches. The key advantage lies in the division of labor, where different microbial species are selected or engineered to perform complementary tasks. In recent years, various SMCs have been evaluated for sewage treatment and the heavy metals remediation [71]. The cocultivation of microalgae and fungi not only treats sewage but also facilitates the easy harvesting of microalgae to produce valuable bioproducts under a circular bioeconomy approach [72]. For example, large-scale algal biotechnology applications were proposed by utilizing algae-fungi consortia for the treatment of anaerobically digested swine lagoon wastewater. It was observed that the total lipid content was also improved. The bioremediation efficiencies of TP, TN, and COD were highest when starch sewage was treated with algae-fungi consortia [73].

Among different SMCs, bacteria-algae consortia developed from activated sludge are mainly studied for wastewater treatment [74]. Microalgae can switch their metabolism from autotrophic to heterotrophic depending on the availability of substrate in the environment. Therefore, microalgae are widely recognized as promising candidates for constructing SMCs for sewage treatment. In this consortia, microalgae and bacteria form a complex symbiotic relationship where bacteria break down complex compounds into simpler organic substances and algae utilize that for growth [75]. Meanwhile, the secretions from algae species serve as primary carbon sources for bacterial growth. In this symbiosis, bacteria mineralize organic matter, providing CO_2_ and nutrients for algae, while the algae produce oxygen that sustains aerobic bacterial metabolism. Several studies have demonstrated that SMC efficiently degrade organic pollutants, nutrients, and heavy metals in various wastewater systems (Table 2). Among these, *Bacillus amyloliquefaciens* has exhibited remarkable potential in removing tetracycline antibiotics and organic contaminants [76]. This emphasizes its significance in multi-species consortia for advanced wastewater treatment. The high remediation efficiencies reported in Table 2 are fundamentally driven by structured microbial interactions and a division of labor that single strains cannot replicate. For example, in algae-bacteria consortia, a clear division of metabolic tasks is established, where heterotrophic bacteria mainly break down complex organic pollutants (reducing COD/BOD), while photoautotrophic microalgae concentrate on nutrient uptake (N, P) and oxygen generation. This relationship is based on obligate cross-feeding, where algae fix CO_2_ released by bacterial respiration, and the O_2_ produced by algal photosynthesis powers aerobic bacterial metabolism. In more complex consortia involving fungi, a spatial division of labor occurs, where fungal mycelia create biofilms that trap particulate matter and heavy metals, increasing their bioavailability for bacterial degradation. These examples illustrate that SMCs are not merely mixtures of microbes but are engineered ecosystems where the collective function emerges from precisely managed interactions and resource exchanges.

As the high metal concentrations could lead to toxicity in algae, SMCs detoxify metals in sewage more efficiently. The growth of microalgae results in the secretion of substances with metal chelation properties, which increase the pH of the environment and allow the heavy metals to precipitate. These precipitates are then easily digested by associated bacteria [77]. One study reported that when algae-bacteria consortia were used, the arsenic and phosphorus removal was highest under varying conditions compared to other systems [78]. It was studied that algae-bacteria consortia removed almost 54% Sulfamethoxazole (SMX) from sewage [79]. This bioremediation potential was found to be linked to symbiotic bioremediation by bacteria that utilize oxygen released during the photosynthesis of microalgae. This synthetic symbiosis not only enhances pollutant removal but also reduces the energy input required for aeration, a major cost in conventional treatment. Another study observed that using microalgae-bacteria consortia, 100% ammonium and 90% COD were removed in a photo-membrane bioreactor for wastewater treatment [80]. Similarly, [81] reported that Chlorella vulgaris and Pseudomonas putida can remove ammonium and phosphate from sewage. They also noted that COD removal was greater in synthetic algae-bacteria consortia than in other systems. The experiments confirmed that the nutrient absorption ability of *P. putida* was improved in the consortia. Though different SMCs could be used for sewage treatment and heavy metals degradation, the practical application of SMCs is limited [82].

**Table 2:** Overview of different microbial consortia and their wastewater bioremediation efficiencies.

Microbial Consortia	Pollutants Targeted	Wastewater	Pollutant Removal Efficiency	References
*Chlorella vulgaris*, *Bacillus licheniformis*	COD, total dissolved phosphorus, total dissolved nitrogen	Municipal wastewater	86.6% COD, 80.3% phosphorus, and 88.9% nitrogen removal	[83]
*A. flavus*, *Fusarium oxysporium*	COD	Textile wastewater	77.6% COD removal	[84]
*Chlorella vulgaris*, *Rhizobium* sp.	TOC, total nitrogen, total phosphate	Synthetic wastewater	49.5 TOC, 55.7% TN, and 95.6 TP	[85]
*Bacillus pumilus*, *Bacillus subtilis*, *Bacillus coagulans, Nitrosomonas* sp., and *Pseudomonas putida*	BOD, COD, TSS, Ammonia	wastewater origin wastewater treatment plant (WWTP)	BOD 71.93%, 64.30% COD, TSS 94.85%, and 88.58% ammonia removal	[86]
*Chlorella* sp., *Heterotrophic bacteria*	COD, Total Nitrogen	Municipal wastewater	86.0% COD and 97.0% Total Nitrogen removal	[87]
*A. fumigatus*, *A. terreus*, *Paenibacillus dendritiformis*	Cadmium	Industrial wastewater	95% removal of Cd	[88]
*Genus pseudomonas* and *Acinetobacter*	pharmaceuticals paroxetine (Prx) and bezafibrate (Bzf)	Activated sludge from WWTP	>97% Prx and Bzf removal	[89]
*Bacillus clausii* and *Bacillus amyloliquefaciens*	Tetracycline antibiotics (TCs)	Laboratory	76.6% removal of TCs by *B. clausii* and 88.9% removal of TCs by *B. amyloliquefaciens*	[76]

## 5. Synthetic Microbial Consortia in PET Degradation

The application of SMCs to PET degradation represents a paradigm shift from relying on single microbial strains or undefined natural consortia. This shift is driven by distinct mechanistic and operational advantages that SMCs offer. Single-strain systems often struggle with the immense metabolic burden of expressing the full suite of enzymes required for complete PET mineralization (e.g., PETase, MHETase, TPA/EG catabolism), leading to suboptimal performance and genetic instability. SMCs overcome this through a deliberate division of labor, distributing these specialized tasks across different populations. This strategy not only alleviates individual metabolic load but also prevents product inhibition, where the accumulation of intermediates, such as MHET, can slow the initial hydrolysis. Furthermore, SMCs can be engineered for synthetic mutualism through cross-feeding, creating stable, cooperative communities that are more resilient and efficient than the sum of their parts.

**Figure 2:**
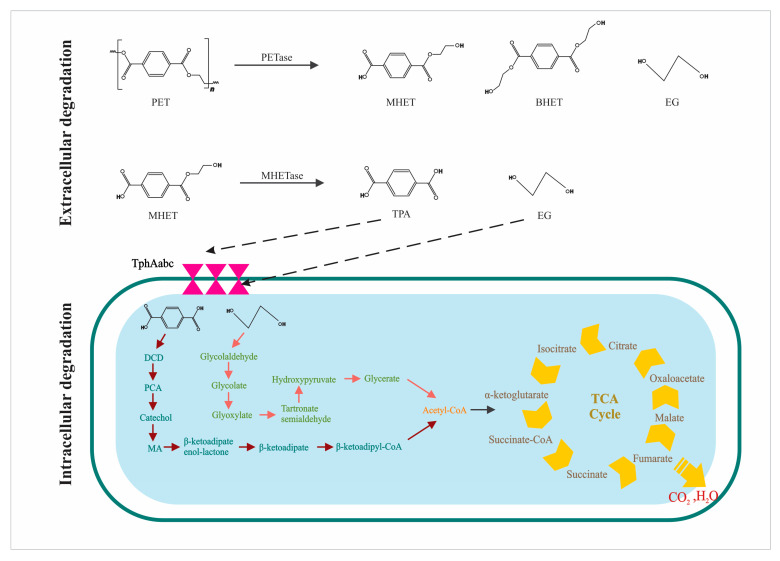
Plastic degradation pathway through PETase and MHETase.

This foundational principle is demonstrated by SMCs containing *Ideonella sakaiensis*, which have shown promising bioremediation potential. *I. sakaiensis* produces two key hydrolytic enzymes (PETase and MHETase) that catalyze the breakdown of PET into its monomers. The wild-type *I. sakaiensis* strain exhibits a PET degradation rate of approximately 0.13 mg cm^−2^ day^−1^ at 30 °C on low-crystallinity film [90]. PETase initially binds to the PET surface using its hydrophobic surface that contains a substrate-binding cleft [91]. A nicking step and a terminal digestion step follow this binding. These steps result in the formation of BHET, MHET, TPA, and EG monomers. BHET can be further degraded into MHET and EG [25]. While the degradation rate of wild-type *I. sakaiensis* is moderate, the engineered cutinases, such as the leaf-branch compost cutinase (LCC), achieve degradation rates exceeding 100 mg cm^−2^ day^−1^ at thermophilic temperatures (65–72 °C) [92]. The detailed process of PET degradation is shown in Figure 2, where PET is degraded into bis(2-hydroxyethyl) terephthalate (BHET), monohydroxyethyl terephthalate (MHET), terephthalate (TPA), and ethylene glycol (EG). In a study, it was found that 78% of the PAHs were degraded efficiently within 70 days by using the bacterial-fungal consortia [93], compared to a single microbe. Another study reported the construction and comparison of different SMCs with varying numbers of species to degrade the PET film. A two-species SMC was constructed that produces PETase and MHETase to degrade the PET film. 13.6% loss of PET film weight within one week [94]. It was observed using a two-species SMC. Further experiments revealed 17.6% weight loss with three-species and 23.2% with four-species SMCs. The increase in degradation efficiency from 13.6% (two-species) to 23.2% (four-species) results from more refined enzymatic cooperation and metabolic division of labor. A simple two-species consortium might feature one strain producing PETase and another producing MHETase. However, in more advanced designs, the labor is further divided where a primary degrader initiates hydrolysis, a secondary consumer specializes in the efficient uptake and metabolism of TPA, and a scavenger strain consumes ethylene glycol (EG). This partitioning prevents the accumulation of inhibitory intermediates (e.g., TPA) and transforms them into biomass, effectively pulling the reaction equilibrium towards further degradation. The stability of this system is often enforced through syntrophic cross-feeding, where the primary degrader depends on vitamins or amino acids produced by the monomer-consuming partners, creating a mandatory mutualism that prevents the collapse of the consortium. This illustrates how SMCs are engineered to function as a coordinated multicellular ‘bioreactor’ for plastic mineralization.

Apart from bacterial-centered SMCs, fungi can also play an essential role in the degradation of plastics. This process begins with the attachment of hyphae to plastic surfaces to create space for other microbes to adhere. After attaching, fungi start converting the plastic polymers into smaller monomers with the help of extracellular enzymes [95]. Major enzymes involved in the process are classified into extracellular and intracellular enzymes. These enzymes depolymerize the plastic polymers into oligomers, dimers, and monomers. These monomers then undergo a series of enzymatic reactions within the cells to form oxidative metabolites. Then the plastic degradation process is completed by other microbes within SMCs. This spatial and metabolic division of labor prevents enzymatic interference and increases the overall degradation surface area. Table 3 shows the bioremediation of plastic using different microbial consortia and the key enzymes involved in the degradation of PET and other plastics. In a study, the ability of *B. subtilis*, *F. oxysporum*, *A. alternata*, and *P. alloputida* in SMCs to degrade LDPE, LLDPE, and PET was analyzed against different SMCs [96]. Furthermore, the biochemical interactions of these SMCs with the plastics were confirmed through FTIR analysis. This analysis revealed the formation of hydroxy and amino groups on the surfaces of PET. Studies have confirmed that SMCs offer scalable and sustainable solutions for plastic degradation; however, further research is required to enhance the efficiency of these processes.

## 6. Advances in Genetic Engineering for Improved Bioremediation

In recent years, genome editing technologies have emerged as powerful tools for enhancing bioremediation, enabling the modification of microorganisms for improved wastewater treatment and plastic degradation. These technologies modify DNA sequences at targeted sites through nuclease-mediated site-specific DNA breaks to produce transformed DNA sequences in microbes that express specific genes associated with microplastic breakdown [104]. Among various genome editing technologies, CRISPR-Cas9 has gained attention due to its accuracy, efficiency, versatility in implementation, and lower execution cost [105]. CRISPR systems have facilitated the enhancement of bioremediation for wastewater and plastics by introducing genes that encode enzymes involved in plastic degradation. This also assists in bioaccumulation, complexation, volatilization, and degradation processes in SMCs [106]. For example, *P. aeruginosa* was genetically engineered to accumulate microplastics in its biofilm by increasing the formation of sticky exopolymer substances [107]. Notably, while this modification enhances pollutant capture, it does not inherently eliminate the organism’s potential pathogenicity as an opportunistic pathogen. However, complementary genetic engineering approaches, such as targeted genome reductions using CRISPR, can attenuate virulence factors in *P. aeruginosa*, resulting in hypo-virulent strains that maintain bioremediation efficacy while improving biosafety for environmental release. Comprehensive risk assessments and regulatory compliance are essential to ensure no unintended pathogenic risks arise from such engineered microbes. These advancements show potential for enzyme-based plastic recycling and improving the efficiency of wastewater treatment through SMCs.

The mechanism of CRISPR-Cas9 involves the introduction of specific guide RNA (gRNA) sequences that target the desired genetic regions within microbes [108]. The insertion or deletion of specific genetic material is then carried out by the Cas9 enzyme to cut the targeted DNA at desired regions. Cas9 can be easily programmed to target any new site by altering its guiding RNA sequence [109]. This approach enhances the ability of microbes to produce enzymes that can break down PET and other plastic polymers. The CRISPR-based techniques can easily modify bacteria and fungi to enhance their ability to degrade plastic [22]. Crucially, this precision enables the engineering of strains not only for enhanced enzyme production, but also for resilience to the volatile conditions of bioremediation sites. For example, genes regulating the production of heat-shock proteins (e.g., rpoH) or chaperones can be overexpressed to improve consortium survival under the temperature fluctuations typical of outdoor bioreactors. Similarly, genes for acid-tolerance (e.g., glutamate decarboxylase systems) or alkali-tolerance can be edited to maintain enzymatic activity across a wide pH range, which is critical as PET hydrolysis can acidify the local environment. A study evaluated that CRISPR-based transposon technology can be used for the precise and site-specific integration Lpp promoter and signal peptide genes into the genome of *E. coli* for PET hydrolysis [110]. Similarly, CRISPR can combine PETase with MHETase or laccases in E. coli to improve the substrate affinity, thermal stability, and degradation rates under industrial conditions [111]. For example, in *E. coli*, a modified PETase was pooled with MHETase to create a bacterial enzyme cascade reaction system for PET degradation [112]. These engineered strains can rapidly degrade PET, even under stress conditions such as elevated temperatures or varying pH levels.

The CRISPR-Cas9 has also been widely used to improve strain tolerance against harsh environments for enhanced microalgal-based wastewater treatment [23]. It increases the metabolite production in algal strains that degrade pollutants in wastewater. Several genes can be engineered to enhance algal potential for wastewater bioremediation using CRISPR-Cas9 technology. The primary issues associated with utilizing the CRISPR-Cas9 technology include gene expression efficiency, stability, and the success of transforming accurate genetic information. However, advances in CRISPR-Cas9 technologies are opening significant opportunities for efficient and controlled wastewater treatment [113]. For example, the genes from the *S. pyogenes* strain and the *P. putida* strain responsible for chromium removal can be inserted and expressed for a pilot-scale bioremediation process [114]. Similarly, the S-adenosylmethionine methyltransferase gene from *Cyanidioschyzon merolae* can be expressed in *B. subtilis* to methylate the organic arsenic [115]. These findings suggest that the CRISPR-Cas9 system could boost the efficiency of wastewater treatment. It provides the precise accuracy, high editing efficiency, multiplex knock-in or knock-out capability, low cost, and a quick cycle for producing bioactive molecules [116]. The CRISPR-Cas9 system can be combined with metabolic engineering to uncover metabolic pathways in microalgae and allow the precise control and high-yield production of desired products [117]. Such genetically enhanced microalgal bioremediation strategies can effectively remove wastewater contaminants.

## 7. Advances in Machine Learning for Modeling and Optimization

Machine learning (ML) has emerged as a powerful approach to enhance the design and functionality of SMCs for the degradation of PET and wastewater treatment. The integration of ML algorithms into microbial ecology has enabled predictive modeling of community dynamics and treatment outcomes. These tools combine microbial data, physicochemical data, and environmental data to simulate system behavior and predict the performance of the process [118]. ML algorithms, such as artificial neural networks (ANNs), random forests (RFs), support vector machines (SVMs), and convolutional neural networks (CNNs), have improved the predictive modeling efficiency of microbial dynamics and treatment performance [119].

A significant strength of ML is its capacity to model the complex, nonlinear relationships between critical environmental parameters and consortium performance. Factors such as pH, temperature, salinity, and initial pollutant concentration profoundly influence microbial activity and enzymatic function. ML models, particularly ensemble methods like Random Forests and Gradient Boosting Machines, are highly effective for identifying which of these parameters are most influential and for predicting SMC stability and degradation rates under such fluctuating conditions. Furthermore, Artificial Neural Networks are well-suited for integrating multi-omics data with real-time environmental sensor data to forecast community behavior. For process optimization, Bayesian Optimization provides a powerful strategy for efficiently navigating the vast experimental space of strain ratios and growth conditions to identify optimal parameters with minimal experimental runs.

In PET degradation, ML models can help in optimizing the enzymes for enhanced performance and stability. ML-guided directed evolution helps to modify the PETase to have a higher optimal temperature (Topt) for efficient degradation of PET [120]. To achieve this, Logistic Regression, Linear Regression, and Random Forest ML models were trained with high accuracy to predict Topt [121]. This algorithm generated hundreds of mutants of PETase, and the mutants with the highest Topt were selected using Random Forest. A new mutant PETase with Topt of 71.38 °C was produced after 1000 iterations. Additionally, a new mutant enzyme was created after 29 iterations at a Temperature of 61.3 °C. The researchers can optimize additional enzymes for improved efficiency by using this approach and novel algorithm.

ML models also overcome the limitations of traditional wastewater treatment technologies and predict uncertain performance outcomes. In complex environmental systems, ML models provide effective results for real-time monitoring, environmental parameter optimization, uncertainty prediction, and fault detection [122]. ML models can analyze multi-dimensional data to monitor process parameters for improved removal of organic and inorganic pollutants in wastewater [49]. It is suggested that ML models offer several advantages in optimizing treatment processes from a wastewater treatment plant (WWTP). These models can predict plant effluent quality parameters and develop the complex and variable behavior of the treatment process. It improves the overall operational control and efficiency to enhance the predictive capabilities of plant systems [123]. ML models can help optimize complex and nonlinear relationships that characterize treatment processes under varying parameters. This ensures better prediction accuracy, which is required in the decision-making process. These models reduce the environmental impact of the wastewater treatment process by improving the design of the WWTP [124]. ANN models are widely used in the prediction of wastewater quality parameters (pH, Electrical Conductivity, and salinity) with high accuracy. This was also supported by other researchers [125], who successfully applied ANNs in wastewater treatment optimization. These advances indicate a pathway toward more sustainable and scalable solutions for plastic degradation and wastewater remediation. However, ML models can predict, control, and optimize bioremediation processes, but ML is still limited to prediction only. Further research is needed to explore the potential of ML models in these processes fully.

## 8. Challenges in SMC Implementations

Despite the considerable promise demonstrated in laboratory studies, the translation of SMCs from controlled environments to real-world, large-scale bioremediation faces significant scientific, technical, and economic hurdles. A critical analysis of these limitations is essential for guiding future research.

### 8.1. Ecological Stability and Biosafety Concerns

A primary challenge is ensuring the long-term stability and predictable function of SMCs upon introduction into complex, open environments. Issues of ecological competition from indigenous microbiota can lead to the rapid displacement of engineered strains. Furthermore, horizontal gene transfer from genetically modified organisms of the consortium to environmental microorganisms raises valid biosafety concerns regarding the uncontrolled spread of engineered traits [126]. Strategies to mitigate these risks are under development, including the engineering of auxotrophies (dependence on synthetic nutrients not found in nature) and kill switches triggered by environmental signals to prevent persistence outside the target site [127,128]. The potential pathogenicity of some candidate strains (e.g., *Bacillus cereus*, *Pseudomonas aeruginosa*) must be rigorously assessed and addressed through attenuation before any field application.

### 8.2. Scalability and Process Engineering

Scaling up SMCs from laboratory bioreactors to industrial or environmental scales introduces profound challenges. Mass transfer limitations can prevent nutrients, signals, and substrates from reaching all consortium members evenly in large volumes or soil matrices [129]. The metabolic burden of expressing heterologous enzymes (e.g., PETase) can reduce fitness and lead to the collapse of the consortium over time. Furthermore, maintaining the optimal stochastic ratio of different populations within the SMC is difficult in dynamic, non-sterile conditions. Advanced bioreactor designs that incorporate spatial compartmentalization and continuous monitoring systems will be crucial to overcome these barriers [130].

### 8.3. Economic Viability and Regulatory Hurdles

The cost of designing, constructing, and maintaining robust SMCs can be prohibitive. A rigorous Techno-Economic Analysis (TEA) is needed to compare SMC-based bioremediation against established physicochemical methods. The economic model could be improved by coupling remediation with the production of value-added bioproducts (e.g., biofuels, bioplastics) from waste streams, creating a circular economy incentive. Finally, the regulatory pathway for the deliberate release of genetically engineered SMCs remains undefined in most jurisdictions, creating uncertainty and posing a significant barrier to commercial adoption [131].

## 9. Conclusions and Future Research Directions

This review provides an in-depth exploration of the molecular principles governing the design and application of synthetic microbial consortia (SMC) for the sustainable bioremediation of wastewater and polyethylene terephthalate (PET). The paper highlighted how the integration of different microbial species, such as bacteria, fungi, and microalgae, can enhance pollutant degradation through synergies, division of labor, and efficient enzyme activation. In addition, the paper highlights the application of advanced molecular tools such as CRISPR-Cas9 and Machine Learning (ML) in building and optimizing these microbial communities for precision pollutant removal and enhancing environmental adaptation. However, the translation of these promising laboratory concepts into robust, real-world applications presents a set of complex interdisciplinary challenges. Overcoming the current limitations in stability, scalability, and economic viability requires a concerted and strategic research effort.

Future research should therefore focus on the rational design of highly stable and modular SMCs using integrated synthetic biology and computational modeling approaches. The development of high-throughput screening systems, multi-omics integration (genomics, transcriptomics, proteomics, and metabolomics), and AI-driven data analysis can further accelerate the discovery of novel microbial interactions and degradation pathways. CRISPR-based multiplex editing can be applied to create synthetic consortia with coordinated regulatory networks, improved substrate affinity, and enhanced tolerance to environmental stresses. Moreover, coupling SMCs with bio-electrochemical systems, membrane bioreactors, and photobioreactors can enhance degradation efficiency and enable simultaneous resource recovery, thereby contributing to a circular bioeconomy. From an environmental perspective, life cycle assessment (LCA) and techno-economic analysis (TEA) are essential for evaluating the feasibility, cost-effectiveness, and sustainability of SMC-based technologies. Interdisciplinary collaboration between microbiologists, biotechnologists, and environmental engineers will be crucial for scaling up these systems from laboratory studies to pilot and industrial applications. In parallel, policy development, risk assessment, and public awareness will play critical roles in ensuring safe and responsible implementation.

## Figures and Tables

**Table 1:** Comparative advantages of bacteria, fungi, and microalgae in bioremediation of wastewater and PET plastics.

Microbial Group	Key Mechanisms	Advantages	Limitations	Relevance to Bioremediation	Examples/References
Bacteria	Enzymatic hydrolysis (e.g., PETase, cutinases); biosorption; metabolic versatility	Rapid growth; high specificity for organic pollutants; effective in heavy metal removal	Sensitive to environmental stress; potential for incomplete degradation	Initial breakdown of PET and wastewater organics; symbiotic roles in consortia	*Ideonella sakaiensis* for PET [25], *Bacillus cereus* for metals [31,48]
Fungi	Extracellular enzymes (e.g., laccases, peroxidases); hyphal penetration; oxidation	Degrades recalcitrant compounds; biofilm formation; tolerant to toxins	Slower growth; nutrient competition	Long-term degradation of hydrophobic plastics; micropollutant oxidation	*Phanerochaete chrysosporium* for organics [35], *Aspergillus* for PET [34]
Microalgae	Biosorption; bioaccumulation; photosynthesis; enzyme expression (e.g., engineered PETase)	Nutrient recycling; oxygen production; sustainable with low inputs	Light-dependent; slower polymer degradation	Nutrient and metal removal; value-added products; enhances consortia oxygen levels	*Chlorella vulgaris* for nutrients [41], Engineered *Chlamydomonas* for PET [42,48]

**Table 3:** Overview of the degradation of different plastics using microbial consortia.

Microbial Consortia	Enzymes	Pollutants	Bioremediation Efficiency	References
*PET*				
*Rhodococcus jostii*, *Bacillus subtilis*	PETase and MHETase	PET	31.2% weight loss in 60 days	[94]
*Pseudomonas putida*, *Bacillus subtilis*	PETase	PET	23.2% PET weight loss with four-species consortium	[7]
*Rhodococcus*, *Pseudomonas putida*, and two metabolically engineered *Bacillus subtilis* species	PETase and MHETase	PET	23.2% weight loss in 7 days	[97]
*Rhodococcus biphenylivorans*, *Burkholderia* sp.	BHET hydrolase (BetH)	BHET (PET intermediate)	95% degradation of BHET in 18 h	[7]
*PS*				
*Stenotrophomonas*, *maltophilia*, *Bacillus velezensis*	kynurenine 3-monooxygenase (Kmo) and 4-hydroxyphenylpyruvate dioxygenase (Hpd)	PS	43.5% weight loss in 60 days	[98]
*PAHs*				
*Rhodococcus* sp., *Acinetobacter* sp., and *Pseudomonas* sp.	dioxygenases and monooxygenases	PAHs	100% degradation 4 weeks	[99]
*PE*				
*Acinetobacter* sp., *Bacillus* sp.	laccase-like multi-copper oxidase (abMCO)	PE	43.5% weight loss in 60 days	[100]
*Enterobacter* sp. *bengaluru-btdsce01*, *Enterobacter* sp. *bengaluru-btdsce02*, and *Pantoea* sp. *bengaluru-* *btdsce03*	Alkane hydroxylase, Hydrolases	PE	81% weight loss in 120 days	[101]
*Bacillus vallismortis*, *Pseudomonas protegens*, *Stenotrophomonas* sp. and *Paenibacillus* sp.	oxidoreductase and hydrolase enzyme	LDPE and HDPE films	75.0% weight loss in LDPE film and 60.0% weight loss in HDPE film	[102]
*PP*				
*Bacillus* and *Pseudomonas*	laccases and alkane hydroxylase enzymes	PP	1.9% weight loss	[103]
*HDPE*				
*Bacillus* sp., *Pseudomonas* sp.	laccase-like multi-copper oxidase (abMCO enzyme)	HDPE	23.14% weight loss in 4 weeks	[100]

## Data Availability

No new data were created or analyzed in this study. Data sharing is not applicable to this article.

## Abbreviations

The following abbreviations are used in this manuscript.

SMC	Synthetic Microbial Consortia
SMCs	Synthetic Microbial Consortia Systems
WWTP	Wastewater Treatment Plant
PET	Polyethylene Terephthalate
QS	Quorum Sensing
ML	Machine Learning
COD	Chemical Oxygen Demand
TPA	Terephthalic Acid
EG	Ethylene Glycol
BHET	Bis(2-hydroxyethyl) Terephthalate
MHET	Mono(2-hydroxyethyl) Terephthalate
